# Identification of an IL-22-Dependent Gene Signature as a Pharmacodynamic Biomarker

**DOI:** 10.3390/ijms22158205

**Published:** 2021-07-30

**Authors:** Julie Rae, Jason Hackney, Kevin Huang, Mary Keir, Ann Herman

**Affiliations:** 1 OMNI Biomarker Development, Genentech Inc., 1 DNA Way, South San Francisco, CA 94080, USA; herman.ann@gene.com; 2 Bioinformatics, Genentech Inc., 1 DNA Way, South San Francisco, CA 94080, USA; hackney.jason@gene.com (J.H.); huang.kevin@gene.com (K.H.); 3 OMNI Biomarker Discovery, Genentech Inc., 1 DNA Way, South San Francisco, CA 94080, USA; keir.mary@gene.com

**Keywords:** IL-22, pharmacodynamic biomarker, IL-1β, IBD, antimicrobial, epithelial repair, gene expression

## Abstract

Interleukin-22 (IL-22) plays a role in epithelial barrier function and repair, and may provide benefits in conditions like inflammatory bowel disease. However, limited human data are available to assess the clinical effect of IL-22 administration. This study used a human intestinal cell line to identify an IL-22-dependent gene signature that could serve as a pharmacodynamic biomarker for IL-22 therapy. The response to IL-22Fc (UTTR1147A, an Fc-stabilized version of IL-22) was assessed in HT-29 cells by microarray, and the selected responsive genes were confirmed by qPCR. HT-29 cells demonstrated dose-dependent increases in STAT3 phosphorylation and multiple gene expression changes in response to UTTR1147A. Genes were selected that were upregulated by UTTR1147A, but to a lesser extent by IL-6, which also signals via STAT3. IL-1R1 was highly upregulated by UTTR1147A, and differential gene expression patterns were observed in response to IL-22Fc in the presence of IL-1β. An IL-22-dependent gene signature was identified that could serve as a pharmacodynamic biomarker in intestinal biopsies to support the clinical development of an IL-22 therapeutic. The differential gene expression pattern in the presence of IL-1β suggests that an inflammatory cytokine milieu in the disease setting could influence the clinical responses to IL-22.

## 1. Introduction

Interleukin-22 (IL-22) has been shown to promote mucosal healing, and to repair epithelial tissue in mouse models, highlighting its potential as a therapeutic agent for diseases with compromised barrier function, including inflammatory bowel disease (IBD). IL-22 is produced by T cells (Th1, Th17, Th22, Tc17, Tc22), γδ T cells, or innate lymphoid cells (ILCs) such as lymphotoxin tissue-inducer (LTi) cells and NK-like or ROR γt+ILCs [1]. In mice, Th17 cells are a major source of IL-22; however, in humans the link between these two cytokines is less pronounced, with IL-22 being produced in the absence of IL-17 by Th22 and Th1 cells [2]. The role of IL-22 in inflammation is influenced by the tissue microenvironment and interactions with other components of the immune system [3]. While the cellular source of IL-22 may differ depending on the model, the activation of the IL-22 pathway has shown benefit in both ulcerative colitis (UC) and Crohn’s disease (CD) in mice [4,5,6,7]. IL-22 is an IL-10 family cytokine that signals through Janus kinases and STAT molecules, primarily STAT3, through a heterodimeric receptor comprised of the epithelial-restricted IL22R alpha (IL22Rα) chain and the ubiquitously expressed IL10R beta (IL10Rβ) chain [8]. IL22Rα is limited to intestinal epithelial cells, hepatocytes, keratinocytes and other epithelial tissues [9]. IL-22 activates three major mitogen-activated protein kinase (MAPK) pathways [9,10,11,12,13], and IL-22-induced cell proliferation has been shown to be regulated by the phosphoinositide 3-kinase (PI3K)/protein Kinase B (akt)/mTOR (mammalian target of rapamycin) cascade in human keratinocytes and fibroblast-like synoviocytes [14].

The role of IL-22 in barrier function and defense is evident from animal models of epithelial repair and protection from pathogens. IL-22 has demonstrated efficacy in animal models such as *C. rodentium* infection [15], pneumonitis [16] and hepatitis [17], as well as the promotion of wound healing in diabetic *db/db* mice [18]. IL-22 has also been shown to be protective in colitis [19,20], including the DSS-induced colitis model using IL-22 or the stabilized IL-22Fc fusion protein UTTR1147A used in the experiments described here, and is under clinical development in IBD [21].

A hallmark of IL-22 function in animal studies is the production of antimicrobial proteins (AMP), including S100A7, S100A8, S100A9, β-defensin-2, β-defensin-3, Reg3β and Reg3γ from epithelial cells [22]. The available human IL-22 data include those from the treatment of a human colon cancer cell line, SW403, with IL-22, which induced the upregulation of *REG1A* and *S100A9* (anti-microbial genes related to those observed in the mouse), *SOCS3* (a common STAT3-driven gene), and *DMBT1* (deleted in malignant brain tumors 1) [23], an antibacterial pattern recognition and scavenger receptor also shown to be upregulated by IL-22 in the human HT-29 and DLD-1 cell lines [24]. AMPs protect mucosal surfaces by killing or inhibiting the growth of microbes, thus managing the interface between bacteria and the epithelial layer, supporting the idea that IL-22 links the adaptive immune response and innate immune mechanisms [25]. Moreover, the mRNA levels of *IL-22* and *DMBT1* were correlated in tissue biopsies from healthy subjects and showed enhanced expression in UC colonic biopsies [23]. In addition, several SNPs in *DMBT1* have been shown to be associated with CD susceptibility [24].

The safety profile of the IL-22Fc fusion protein UTTR1147A has been established in multiple species [26], and the pharmacology suggests translatability to clinical use in humans. The pharmacodynamic (PD) biomarkers used in preclinical studies were the serum Reg3β and SAA in rodents, and REG3A, LBP and SAA in cynomolgus monkeys. The clinical development of IL-22 in IBD and other diseases with epithelial injury will benefit from a thorough understanding of its effects in human target tissues. With this in mind, we sought to develop a gene expression signature that could be used in colonic tissue biopsies to support the clinical development of UTTR1147A in UC. As a PD biomarker, a gene signature would need to be highly sensitive to IL-22 stimulation, dose-dependent across a wide range of doses, able to differentiate IL-22 from the other inflammatory cytokines also present under disease conditions, and have available robust primer/probe sets for detection. These characteristics would allow the assessment of the relationship between the pharmacokinetics (PK) and PD at the site of action in the gut. Here, we used the IL-22 responsive HT-29 human intestinal cell line to identify an IL-22-inducible gene signature that could be used as a sensitive PD biomarker in gut mucosa for the clinical translation of UTTR1147A. 

## 2. Results

### 2.1. Characterizing HT-29 Cells 

**Figure 1:**
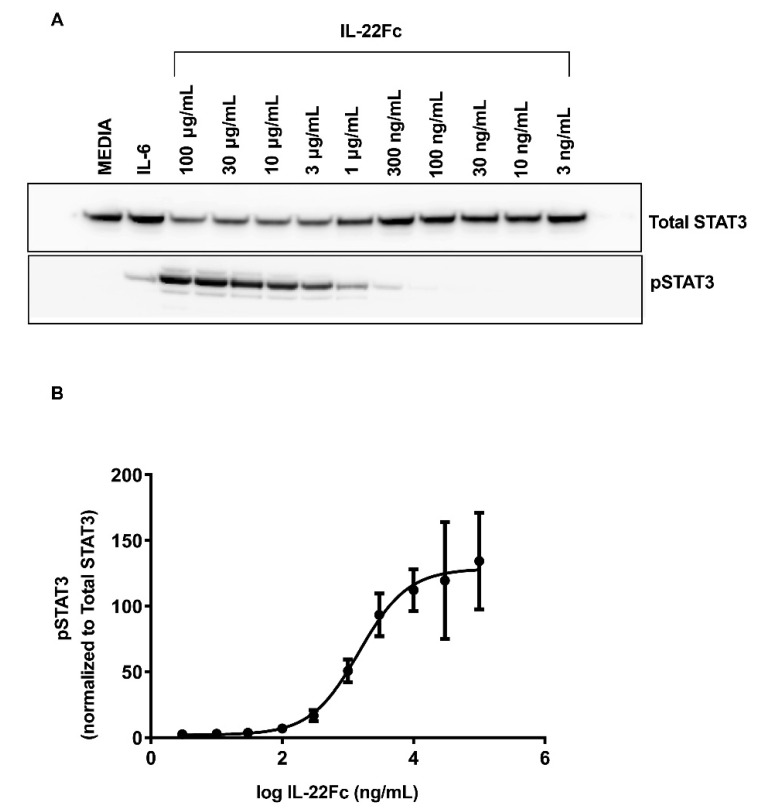
IL-22-mediated STAT3 activation, as measured in HT-29 cells. The HT-29 cells were cultured for 5 days with media, followed by an additional 18 h in serum-free media. Varying concentrations of IL-22 or 25 ng/mL rhIL-6 were added to the cell cultures for 20 min. The cell lysates were analyzed by Western blot. (**A**) Representative Western blot densitometry showing the pSTAT3 and total STAT3. (**B**) Levels of pSTAT3 normalized to STAT3, shown as the mean ± SD of three biological replicates. The EC_50_ is 1.56 μg/mL.

In order to assess the human colon epithelial HT-29 cells for their ability to mimic the in vivo UTTR1147A responses observed in animal models [21], we first looked at their ability to activate the STAT3 pathway upon activation with UTTR1147A. Both IL-22 and IL-6 are known to induce the phosphorylation of STAT3 upon binding to their respective receptors; therefore, IL-6 treatment was used as a positive control for stimulation (Figure 1A). IL-6 stimulation demonstrated increased pSTAT3 levels, as expected. The pSTAT3 levels were also upregulated inHT-29 cells in a dose-dependent manner upon treatment with UTTR1147A, reaching the maximal effect at 30 µg/mL (Figure 1B).

### 2.2. Development of a Specific, Sensitive and Dose-Dependent IL-22 Responsive Gene Signature in HT-29 Cells 

**Figure 2:**
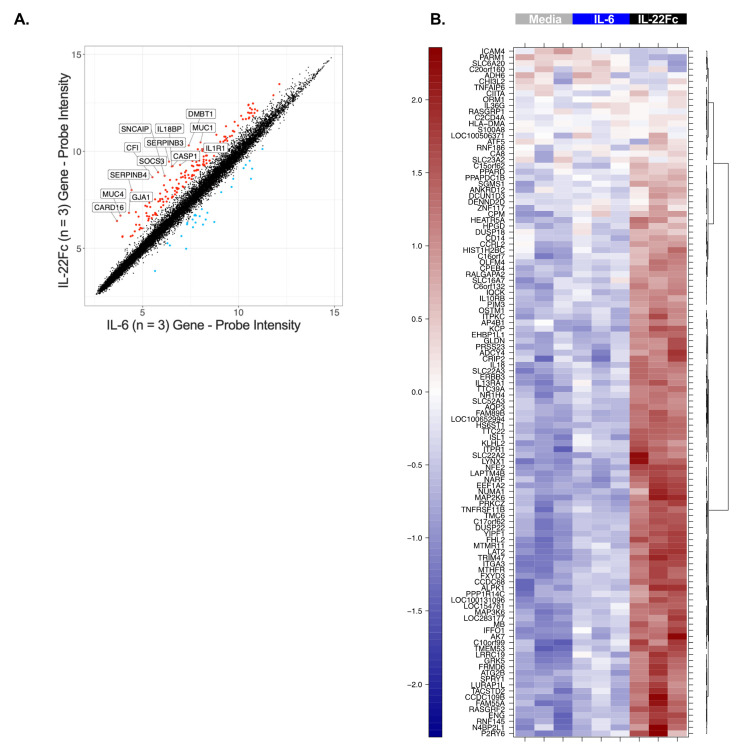
Gene upregulation induced by IL-22 and not IL-6. (**A**) Scatterplot comparing the microarray gene expression from HT-29 cells cultured for 24 h with either UTTR1174A (IL-22Fc) or IL-6 prior to the RNA isolation. The colored points indicate genes with significant differential expression (2.0-fold change, adjusted *p* < 0.05). The labeled points indicate 13 genes with a fivefold change (adjusted *p* < 0.05). (**B**) Heat map from the same experiment using stricter specificity criteria, defined as the union of genes that were at least 1.5-fold upregulated by UTTR1147A, less than 1.2-fold upregulated by IL-6, and had at least a 1.5-fold difference between UTTR1147A and IL-6. Three biological replicates are represented.

**Figure 3:**
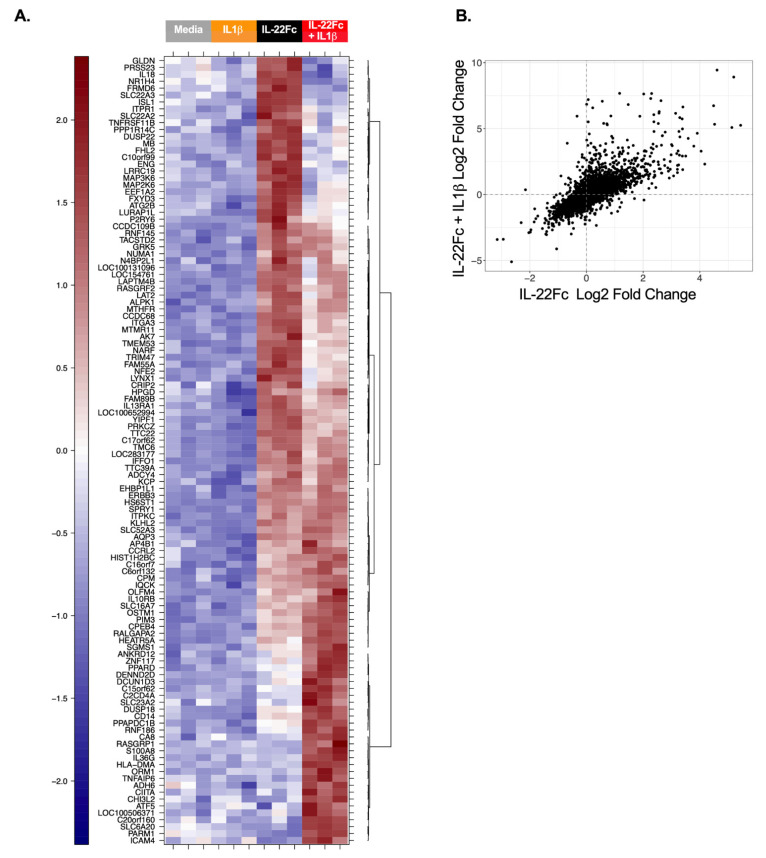
Differential gene expression by IL-22 in the presence of IL-1β. (**A**) Heat map with the microarray gene expression from HT-29 cells cultured for 24 h with or without IL-1β, IL-22Fc or IL-22Fc + IL-1β prior to the RNA isolation. Three biological replicates are represented. (**B**) Scatterplot showing the genes upregulated by IL-22 alone vs. IL-22 + IL-1β.

In order to identify the gene expression changes following IL-22 stimulation, HT-29 cells were cultured with a maximal dose of UTTR1147A (30 µg/mL) for 24 h prior to RNA isolation, followed by microarray analysis, based on the timing of previous gene expression data in mouse colon ex vivo cultures [15]. Three hundred and sixty-six genes were found to be induced ≥2.0-fold in HT-29 cells treated with UTTR1147A with an adjusted *p*-value < 0.05, while 84 genes were repressed >2.0-fold. In order to focus on genes that were specific for IL-22, rather than genes activated via other STAT3-dependent pathways, such as IL-6, we compared the IL-22 gene signature to the set of genes upregulated by IL-6. Thirteen genes were upregulated at least 5.0-fold greater with IL-22 compared to IL-6, and these included *IL1R1*, *DMBT1* and *IL18BP* (Figure 2A). Another sixty-eight genes with greater IL-22 specificity were defined as the union of genes that were at least 1.5-fold upregulated by UTTR1147A, less than 1.2-fold upregulated by IL-6, and had at least a 1.5-fold difference between UTTR1147A and IL-6 (Figure 2B). *IL1R1* was upregulated by UTTR1174A compared to IL-6 (Figure 2A). Therefore, IL-1 may impact the gene expression pattern of IL-22, because *IL-1α* and *IL-1β* are upregulated in IBD [27]. We could demonstrate that the UTTR1174A signature was altered in the presence of IL-1β (Figure 3A) by repeating the same microarray experiment using the co-culture of HT-29 cells with UTTR1147A and IL-1β. The assessment of the genes that are upregulated by UTTR1147A alone or in combination with IL-1β allows the identification of the genes of which the IL-22-dependent expression may be increased in the setting of inflammation. With the combination of UTTR1147A and IL-1β, 58 genes were upregulated ≥1.5-fold. The genes that were at least 1.5-fold upregulated by UTTR1147A + IL-1β, <1.2-fold upregulated by IL-6 + IL-1β, and had at least a 1.5-fold difference between UTTR1147A + IL-1β and IL-6+ IL-1β were selected for further study as a potential PD gene signature (Figure 3B). Twelve genes appeared in both groups (with or without IL-1β). This curated set of 117 genes was further assessed for potential clinical translation using gene expression data from human colon biopsies comparing inflamed to uninflamed tissue, as determined by the endoscopist [28]. Genes that are already highly upregulated in inflamed tissue may not yield a dynamic range that would enable further upregulation by UTTR1147A to be observed; 30 genes were removed for this reason. 

**Figure 4:**
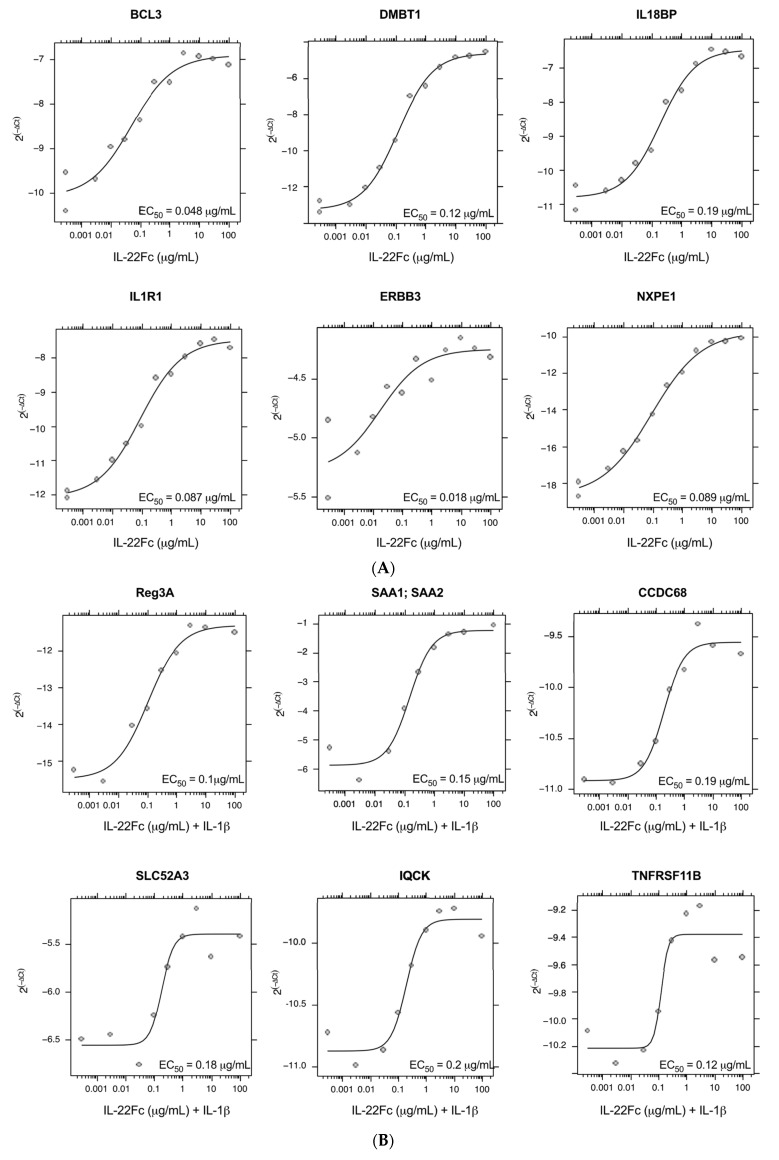
IL-22 dose-responsive genes. (**A**) Representative plots of the genes that were the most dose responsive to IL-22Fc. (**B**) Representative plots of the genes that were the most dose responsive to IL-22Fc+ IL-1β.

Seventy-eight genes from this curated gene set had available robust primer/probe sets and were re-assessed by qPCR (Table 1). An additional eight genes that did not meet the criteria above (enhanced gene expression with IL-22Fc vs IL-6, in the presence or absence of IL-1β) were added back into the qPCR assessment, because they had been shown to have in vivo biological significance with IL-22 treatment or were important in the IL-22 pathway (e.g., *IL22R1* and *IL22RA2*), along with two IL-6-responsive genes (*SBNO2*, *BCL3*) as controls (Table 1) [22,23,29,30]; qPCR confirmation using the same RNA samples as the microarray showed 69/78 genes with an upregulation (> 1.5 FC) by UTTR1147A ± IL-1β. Additional qPCR using the RNA from HT-29 cells treated with multiple doses of UTTR1147A ± IL-1β revealed 38 genes, including IL1R1, which showed dose-dependent induction. (Figure 4). The dose–response curves demonstrated that many of the genes were sensitive to very low levels of UTTR1147A, with 85 detectable at 300 ng/mL, 80 at only 30 ng/mL, and 39 with detectable gene expression with as little as 3 ng/mL IL-22Fc stimulation in the absence (Figure 4A) or presence (Figure 4B) of IL-1β. 

### 2.3. HT-29 Cell Secretion of Antimicrobial Peptides REG3A and SAA Confirms the IL-22/IL-1 Gene Expression Pattern 

**Figure 5:**
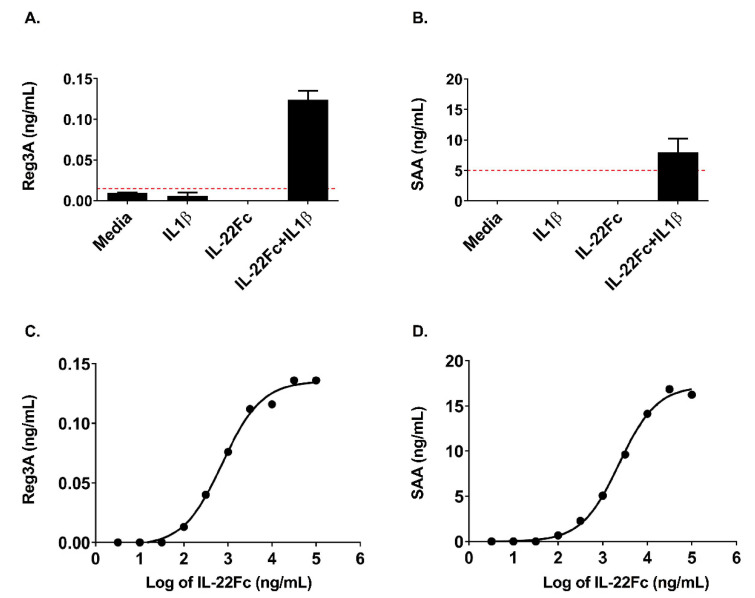
Secretion of acute phase proteins by HT-29 cells requires IL-1β. (**A**) REG3A (Pancrepap), as measured by ELISA, showing detectable levels only with the addition of IL-1β, shown as the mean +/- SD, *n* = 2. The dashed red line indicates the LLOQ of the assay. (**B**) Serum Amyloid A (SAA), as measured by ELISA, showing detectable levels only with the addition of IL-1β, shown as the mean +/- SD, *n* = 2. The dashed red line indicates the LLOQ of the assay. (**C**) REG3A (Pancrepap) representative dose–response curve, as measured by ELISA. The points represent the mean of the duplicate wells, EC_50_ = 2.3 μg/mL. (**D**) Serum Amyloid A (SAA) representative dose–response curve, as measured by ELISA. The points represent the mean of the duplicate wells, EC_50_ = 0.74 μg/mL.

In order to understand whether the IL-22 gene expression data translated to changes at the protein level, we assessed the secretion of REG3A and SAA proteins (used as secreted PD biomarkers in preclinical studies) by HT-29 cells following IL-22Fc stimulation in the presence or absence of IL-1β (Figure 5). REG3A and SAA proteins were produced only when UTTR1147A was used in combination with IL-1β, as is consistent with IL-22 alone and the IL-22+IL-1β gene signatures (Figure 5A,B). Additionally, the increases in the secreted proteins were dose dependent, similar to the gene expression, and sensitive, with REG3A and SAA being produced at doses as low as 100 ng/mL of UTTR1147A, but only in the presence of IL-1β (Figure 5C,D). 

## 3. Discussion

IL-22 is of interest as a therapeutic target due to its role in epithelial protection, barrier function and repair. The IL-22 IgG fusion protein, UTR1147A, is currently in clinical development for diseases with epithelial injuries such as IBD. In addition to the assessment of the nonclinical safety and pre-clinical and translational pharmacology [21,26] of UTR1147A, we describe the identification of a sensitive gene signature in a human intestinal epithelial cell line. For a gene signature to act as a clinically translatable biomarker, it must be in an accessible tissue, highly sensitive to low doses of drug, able to differentiate IL-22 from the inflammatory cytokines also present under disease conditions, and dose-dependent across a range of concentrations. Human intestinal epithelium is an attractive target for biomarker assessment in IBD because it is obtained through biopsies that can be taken during routine endoscopies performed for patient monitoring and efficacy measures. Here, an intestinal epithelial cell line was used to identify an IL-22-induced gene expression signature which might be used as a PD biomarker in human mucosal biopsies from IBD patients treated with UTR1147A. 

In order to generate a specific gene signature, we evaluated the expression of genes following UTR1147A stimulation and filtered out genes that were also highly upregulated by IL-6 (i.e., STAT3-dependent but not IL-22 specific). This approach was selected to deconvolute the contribution of IL-22 as a therapeutic from those of general inflammation signals mediated through pSTAT3. We were able to identify genes enriched for IL-22 response compared to IL-6, albeit not completely exclusive to IL-22, given the close overlap in the genes induced given the cytokines’ shared STAT3 signaling pathway (Figure 2A). The upregulation of IL1R1 by IL-22 in the HT-29 cells was identified in the initial experiments and further explored for its potential to alter the gene expression patterns in the target cells. Previously, HT-29 cells were shown to produce significantly increased levels of CXCL8 protein in the presence of IL-22 and TNF-α or IL-1β compared to those with TNF-α or IL-1β alone [38], where the authors suggest that the angiogenesis-enhancing capacity of CXCL8 may contribute to the role of IL-22 in wound-healing responses. In the current study, the combination of IL-22 and IL-1β generated an altered gene expression pattern compared to IL-22 in the absence of IL-1β (Figure 3). *DMBT1* was identified as a characteristic IL-22-responsive gene in a prior study, and here it was not differentially expressed in the presence or absence of IL-1β, confirming the previous mouse and in vitro human studies [23,39]. However, the pattern observed in the presence of IL-1β was most similar to the ex vivo signatures identified in mice [15], and included the upregulation of anti-microbial peptides such as REG3A and SAA, a hallmark of IL-22 function. The differentiation of Th17 cells via IL-23 or IL-6 is dependent on the presence of IL-1β [40], and IL-1β is also involved in the differentiation of innate T cells and ILCs to IL-22-producing subsets [2]. Therefore, IL-1β is likely to be present in the inflammatory milieu where these cells develop, and may modulate the gene expression response to IL-22 when present. This may suggest that the differential pattern of expression in the presence of IL-1β is relevant in primary epithelium, and that a successful PD biomarker gene signature in the gut should take genes upregulated by IL-22 in the presence or absence of IL-1β into account. Several genes identified from the IL-22 signature, either in the presence or absence of IL-1β, demonstrated high sensitivity to UTTR1147A at low doses and were dose-responsive across a broad range of doses, suggesting that the signature may be useful as a clinically translatable PD biomarker (Figure 4).

Interestingly, some genes upregulated by UTTR1147A in HT-29 human epithelial cells have been reported as having a genetic association to IBD. For example, *Lynx1*, which encodes a member of the Ly-6/neurotoxin gene family, has been shown to be upregulated by IL-22 in murine colons [41]. Using expression quantitative trait loci (eQTL) combined with genome-wide association study (GWAS) patterns in humans, *Lynx1* was associated with Crohn’s disease [31,41]. GWAS analysis has also implicated *CPEB4* as being associated with Crohn’s disease [34,42]. Purinergic signaling is known to regulate several important functions in the gut, and may be important in IBD. Similarly, *P2RY6* has been shown to be downregulated in mucosal biopsies from UC patients [35]. *CDH1*, which encodes E-cadherin, is a strong candidate for UC susceptibility [43] and is the first genetic correlation between UC and colorectal cancer [44]. *DMBT1*, which was upregulated by IL-22Fc and was neutral to the presence of IL-1β, is also involved in IBD, as shown by [23] where *IL-22* and *DMBT1* transcripts were upregulated in mucosal tissues from UC patients, as well as by Deigelmann et al. [24], who identified several *DMBT1* variants associated with CD susceptibility. 

Overall, these data suggest that the IL-22 pathway may be altered based on the presence of an inflammatory cytokine milieu, especially IL-1β in the setting of inflammation. The release of AMPs, a key mechanism of IL-22 protection, may be differentially altered depending on the IL-1 levels. In summary, the PD biomarker gene expression signature identified here may allow improved clinical translation by acting as a sensitive measure of proximal target engagement in IBD patients treated with IL-22Fc, even in the complex environment of the gut.

## 4. Materials and Methods

### 4.1. Cell Culture and Reagents

The human colorectal adenocarcinoma cell line HT-29 was obtained from ATCC (Manassas, VA, USA) and cultured in GIBCO^®^ low-glucose Dulbecco’s modified Eagle Medium (LGDMEM) (Life Technologies, Carlsbad, CA, USA) containing 2 mM *L*-glutamine and 10% FBS at 37 °C in a humidified atmosphere containing 5% CO_2_. The recombinant IL-22Fc fusion protein (also named UTTR1147A, hereafter called “UTTR1147A”) was generated in-house. UTTR1147A is a human interleukin-22 (IL-22) fusion protein that links IL-22 to the Fc portion of human immunoglobulin G4 (IgG4). The Fc portion of the fusion protein incorporates a mutation (N297G) that minimizes the potential for Fc effector function. UTTR1147A is produced in Chinese hamster ovary cells and has a deglycosylated molecular weight of approximately 85 kDa [21], requiring higher concentrations of the fusion protein compared with standard recombinant cytokine IL-22. The human recombinant IL-6 and IL-1β were purchased from Pierce (Invitrogen; Frederick, MD, USA). 

### 4.2. In Vitro Stimulation of HT-29 Cells and the Analysis of the Phospho-STAT3 

The HT-29 cells were plated in 24-well tissue culture plates (Nunc) at 10^5^ cells/well, and cultured at 37 °C. After 5 days, the media were replaced with serum-free media and cultured for an additional 18 h. UTTR1147A or 25 ng/mL rhIL-6 (Invitrogen; Frederick, MD, USA) was added to the culture for 20 min. Following stimulation, the cultures were placed on ice, washed with cold phosphate-buffered saline (PBS), and lysed in 1X RIPA buffer (Millipore, Temecula, CA, USA) containing protease and phosphatase inhibitors (Thermo Fisher Scientific, Rockford, IL; Sigma Aldrich, St. Louis, MO, USA; and Roche, Indianapolis, IN). The protein concentration was determined using the BCA-Pierce^®^ Assay (Calbiochem, Darmstadt, Germany). 

The protein lysates were thawed, and duplicate samples (40 µg/lane) were loaded onto 4−12% tris bis gels (Novex by Life Technologies; Carlsbad, CA, USA), run, and transferred to nitrocellulose membranes. The blots were probed using a STAT3 or pSTAT3-Try705 primary antibody (Cell Signaling, Danvers, MA, USA), followed by a peroxidase conjugated goat anti-rabbit F(ab’)2 secondary antibody (Jackson Immuno Research, West Grove, PA, USA). The blots were imaged using the ImageQuant 4000 and quantified with the ImageQuant software (GE Healthcare Life Sciences; Pittsburgh, PA, USA). The pSTAT3 protein levels were normalized to total a STAT3 protein level for each sample. The concentration required for a 50% increase (EC_50_) in the phosphorylated STAT3 was calculated by performing a non-linear regression curve fit using GraphPad Prism 8 (v.8, GraphPad Software, Inc.; La Jolla, CA, USA).

### 4.3. In Vitro Stimulation of the HT-29 Cells, mRNA Sample Preparation and Microarray Analysis

HT-29 cells cultured for five days were stimulated with cytokines (30 μg/mL for UTTR1147A, 100 ng/mL IL-1β and 25 ng/mL IL-6) for 24 h. Following the stimulations, the cells were lysed, then homogenized using QIAshredder columns (Qiagen, Hilden, Germany), and the RNA was thereafter extracted using the RNeasy kit (Qiagen, Hilden, Germany). The RNA was subjected to microarray analysis using GeneChip^®^ Human Genome U133 Array Plus 2.0 (Affymetrix, Santa Clara, CA, USA). 

### 4.4. Quantitative Real-Time PCR Analysis

A subset of genes curated from the microarray data (described below and listed in Table 1) were used for the qPCR experiments. The same RNA generated for the microarray experiments was used for qPCR, as well as new samples generated using HT-29 cells stimulated with UTTR1147A, with or without IL-1β (100 ng/mL) for 24 h. The UTTR1147A doses ranged from 3 ng/mL to 100 μg/mL, and the RNA was prepared as above. The real-time qPCR was performed using the BioMark gene expression platform (Fluidigm). Briefly, 2 µL total RNA was reverse-transcribed to cDNA and pre-amplified in a single reaction using Superscript III/Platinum Taq (Invitrogen), SUPERase-IN (Ambion, Austin, TX, USA) and 2X reaction mix (Applied Biosystems, Foster City, CA, USA). The pre-amplification reaction was performed at a final dilution of 0.05× for the original Taqman assay (Applied Biosystems) concentration using the following conditions: one cycle at 50 °C for 15 min and one cycle at 70 °C for 2 min, followed by 18 cycles at 95 °C for 15 s, and 60 °C for 4 min. The gene expression of the target genes using the available primer/probe pairs (Thermo Fisher Scientific, Rockford, IL) was measured using a Taqman Universal PCR MasterMix (Applied Biosystems) on the BioMark BMK-M-96.96 platform (Fluidigm, South San Francisco, CA, USA), according to the manufacturer’s instructions. All of the gene expression values were normalized to GUSB expression using the delta Ct method. All of the samples were assayed in triplicate. The dose–response analyses were performed using the drc R package [45]. We used the LL2.4 four-parameter log-logistic function for the fitting of the dose–response curves. The dose–response relationships were considered significant if they had a nominal (unadjusted) *p*-value < 0.05.

### 4.5. Microarray Analyses

The microarray data were processed using the RMA method from the affy R package [46]. We used the limma package for the determination of the differential expression across the conditions [47] using the default settings. The gene selection process was designed to identify genes that were upregulated more by IL-22 than IL-6 (a well-known STAT3 signaling cytokine). A first pass identified the genes which were the most highly upregulated by IL-22 when compared to IL-6 (5-fold or greater), and these included IL1R1 (Figure 2A). We then used the criteria described below to identify additional genes upregulated more by IL-22 than IL-6, as well as by the combination of IL-22 and IL-1β using the following criteria: 

With a fold change (FC) ≥ 1.5 and a false discovery rate (FDR) ≤ 0.001:

- up-regulated in IL-22Fc v. Media, *and*
- up-regulated in IL-22Fc v. IL-6, *and*
- up-regulated in IL-22Fc v. IL-1β

With FC ≥ 1.2 and FDR ≤ 0.2:

- not up-regulated in IL-6 v. Media, *or*
- not up-regulated in IL-1β v. Media, *or*
- not up-regulated in IL-6 + IL-1β v Media

Or:

With fold change (FC) ≥ 1.5 and false discovery rate (FDR) ≤ 0.001:

- up-regulated in IL-22Fc + IL-1β v. Media, *and*
- up-regulated in IL-22Fc + IL-1β v. IL-6 + IL-1β, *and*
- up-regulated in IL-22Fc + IL-1β v. IL-1β

With FC ≥ 1.2 and FDR ≤ 0.2:

- not up-regulated in IL-6 v. Media, *or*
- not up-regulated in IL-1β v. Media, *or*
- not up-regulated in IL-6 + IL-1β v Media

The genes fulfilling these criteria were then ranked using data which looked at the gene expression of inflamed (from UC patients) vs. non-inflamed colon tissue (from healthy controls), with those already upregulated in the inflamed tissue ranked lower [28]. Seventy-eight genes from these analyses were selected for further study, with an additional eight genes included based on previously identified IL-22 biology (e.g., IL-22 receptor genes and the genes of PD biomarkers previously observed in literature [26]), and two additional IL-6-responsive control genes as well (Table 1). 

### 4.6. SAA and REG3A ELISA Assays

The HT-29 cells were plated for 5 days, followed by stimulation after plating with UTTR1147A (3.3 ng/mL to 10 µg/mL) and/or IL-1β (25 ng/mL). After two days, the culture supernatants were removed and frozen at −20 °C. The protein analysis for SAA (Invitrogen; Frederick, MD, USA) and REG3A (Dynabio; Marseille, France) were performed on the thawed supernatants according to the manufacturer’s instructions. 

## Figures and Tables

**Table 1:** Genes upregulated by IL-22Fc.

Gene	Loci *	Gene	Loci *
*ADH6*		***ITPKC***	
***AK7***		***ITPR1***	
*ALPK1*		***KCP***	
***ANKRD12***		***LAPTM4B***	
***AP4B1***		***LAT2***	
***ATF5***		***LURAP1L***	
*ATG2B*		***LYNX1***	**CD** [31]
**BCL3** ^†^		***MAP2K6***	
***CA8***		***MB***	
***CCDC68***		***MTHFR***	
*CCM2L*		*NARF*	
***CCRL2***	**IBD** [32]	***NR1H4***	
***CD14***		*NUMA1*	
*CDH1* ^#^	**UC** [32]	***NXPE1***	**UC** [33]
***CHI3L2***		***ORM1***	
***CIITA***		***OSTM1***	
***CPEB4***	**CD** [34]	***P2RY6***	**UC** [35]
***CPM***		***PARM1***	
***DCUN1D3***		***PPAPDC1B***	
***DENND2D***		***PPARD***	
***DMBT1***	**IBD** [31,34]	***PPP1R14C***	
***EEF1A2***		***PRKCZ***	
*EPCAM* ^#^		***PRSS23***	
***ERBB3***		***RALGAPA2***	
***FHL2***		***RASGRP1***	**CD** [36]
***FRMD6***		***REG3A*^#^**	
***FXYD3***		***REG3G*^#^**	
***GLDN***		*RNF145*	
*HIST1H2BC*		***RNF186***	**UC** [37]
***HLA-DMA***		***S100A8***	
***HS6ST1***		***S100A9*^#^**	
*ICAM4*		***SAA1;SAA2*^#^**	
***IFFO1***		***SAA2*^#^**	
***IL10RB***		***SBNO2*** ^†^	
***IL13RA1***		***SLC16A7***	
***IL18***		***SLC22A2***	
***IL18BP***		***SLC22A3***	
***IL1R1***		***SLC23A2***	
***IL1RAP***		***SLC52A3***	
*IL22RA1* ^#^		***SPRY1***	
*IL22RA2* ^#^		***TMEM53***	
***IL36G***		***TNFAIP6***	
***IQCK***		***TNFRSF11B***	
***ISL1***		***TTC39A***	
***ITGA3***		***YIPF1***	

The gene names in bold show upregulation by UTTR1147A +/- IL-1β, and were confirmed by qPCR. ***^#^*** Genes whichwere included due to their known IL-22 biology. * Published susceptibility loci. ^†^ Control IL-6-responsive genes.

## Data Availability

The microarray data that support the findings of this study are openly available in the Gene Expression Omnibus (GEO) at https://www.ncbi.nlm.nih.gov/geo/, accession number GSE159223, accessed on 8 October 2020.
